# Gendered networks of psychological adaptation under prolonged war

**DOI:** 10.3389/fpubh.2026.1920728

**Published:** 2026-09-08

**Authors:** Liat Hamama, Yaira Hamama-Raz, Orly Sarid

**Affiliations:** 1 School of Social Work, Tel Aviv University, Tel Aviv, Israel; 2 School of Social Work, Ariel University, Ari'el, Israel; 3 The Spitzer Department of Social Work, Goldman Sonnenfeldt School of Sustainability and Climate Change, Ben-Gurion University of the Negev, Be'er Sheva, Israel

**Keywords:** coping, gender, network analysis, psychological adaptation, war

## Abstract

**Introduction:**

Understanding how men and women navigate profound, prolonged adversity is central to human resilience. Drawing on frameworks of masculine norms and emotion regulation, and moving beyond traditional mean-level comparisons, this exploratory, cross-sectional study used a network approach to examine gender differences in the structural organization of traumatic life events, stress symptoms, health-related quality of life, meaning in life, and coping strategies during prolonged war.

**Methods:**

We assessed 313 Israeli adults (Mean age = 43.49, SD = 14.13; 51% women) via self-report questionnaires. Network analyses compared self-identified men and women across structural topology, community organization, centrality, and predictability.

**Results:**

The primary Network Comparison Test detected no statistically significant difference between men and women in overall network structure or global strength. No individual edge or node-centrality difference survived correction for multiple comparisons. Secondary analyses nevertheless identified more localized differences in selected aspects of topology, community organization, bridge connectivity, and predictability. Specifically, the women's network showed higher density and local clustering, and dysfunctional coping differed in its community placement and cross-community connectivity, whereas the men's network showed higher mean node predictability. These secondary findings describe localized features within networks that remained comparable at the global level and should be interpreted as exploratory, cross-sectional evidence.

**Conclusions:**

These structural observations outline suggestive patterns that warrant confirmation in future longitudinal samples, rather than serving as direct bases for clinical protocols. Formulated as hypotheses for future research, the men's integrated networks suggest that single, coping-focused interventions might be associated with broader changes across symptoms. In contrast, the women's modular networks suggest a potential need for multi-targeted approaches incorporating broader social resources. Ultimately, these descriptive findings highlight the value of gender-sensitive frameworks for understanding psychological adaptation to chronic collective threats.

## Introduction

The question of how men and women navigate profound and prolonged adversity is central to understanding human resilience and vulnerability, particularly under conditions of sustained war and armed conflict exposure. It is well-established that such environments generate widespread psychological stress and significantly increase anxiety, depression, and post-traumatic stress disorder (PTSD) symptoms (1–3), disrupting societal and individual well-being (3–6). Heightened vulnerability is robustly linked to the accumulation, frequency, and severity of traumatic events, which can diminish health-related quality of life long after conflicts cease (7–10). Within this context, a critical inquiry remains: Do gender-specific patterns of coping and adaptation persist or fundamentally shift in the face of chronic, ongoing stress? Gender encompasses the social, cultural, and behavioral characteristics linked to gender identities (distinct from biological sex) (11) that deeply shape how individuals interpret and respond to threats. Although gender and sex are conceptually distinct, the current study relies on binary demographic reporting to examine structural differences between self-identified men and women.

Significant differences in stress responses between women and men have been consistently documented in the literature. Women frequently report higher overall levels of psychological distress (1, 3, 12, 13) and lower mental health-related quality of life (9). Comprehensive reviews and meta-analyses indicate that gender differences in responses to stress are highly context-dependent (14–17). Rather than reflecting a consistent vulnerability in either group, these differences may vary according to patterns of exposure, the nature and interpersonal meaning of the stressor, and gender-role socialization. For example, socially threatening events involving status loss may be associated with greater post-traumatic distress among men than among women (18). Although much of this literature focuses on PTSD, the mechanisms it identifies may also inform the broader chronic stress responses examined in the present study, including anxiety, depression, and general well-being.

To explain these patterns, the present study draws on emotion-regulation and gender-role frameworks. Women generally report greater emotional awareness and a broader repertoire of adaptive regulation strategies; however, a stronger tendency toward rumination may increase vulnerability to anxiety and depression under prolonged stress (19, 20). Cumulative strain may also increase reliance on maladaptive strategies that provide short-term emotional relief (21). Men's responses may be shaped by traditional masculine norms emphasizing emotional control, self-reliance, and the concealment of vulnerability (22). Such norms may inhibit emotional disclosure and help-seeking and impede the recognition, expression, and regulation of distress (23). Together, these perspectives suggest that gender-related patterns of adaptation reflect an interaction between the strategies individuals employ and the social norms shaping how distress is experienced, expressed, and managed.

Viewed through these theoretical lenses, coping strategies may reflect how emotion-regulation processes and internalized gender norms are expressed in cognitive, emotional, and behavioral responses to stress. Specific coping efforts encompass emotion-focused, problem-focused, and dysfunctional strategies (24). Women generally report greater use of emotion-focused coping than men (25–27). During crises, dysfunctional coping has been associated with poorer quality of life, whereas problem-focused and emotion-focused strategies have generally been associated with more adaptive outcomes (10, 28).

Related to these theoretical perspectives, meaning in life comprises the subjective experience of life as purposeful (“presence”) and the motivational drive to establish purpose (“search”) (29). It has been conceptualized as a psychological resource in the context of stress and adversity. Some studies have reported that women score higher than men on both the presence of and search for meaning in life (30). In addition, the presence of meaning in life has been more strongly associated with lower levels of depression among women than among men in some samples (31). In contexts of ongoing conflict, meaning in life has been associated with better health-related quality of life and lower levels of depression and anxiety (6, 32, 33). More generally, higher levels of meaning in life have been linked to adaptive self-regulation, preventive health behaviors (34), resilience (5), and adaptive coping, including problem-focused coping (35). Conversely, anxiety and depression have been negatively associated with perceived meaning in life (31, 36).

The current Israeli context provides an interesting case in which to examine whether these gender-specific structural patterns hold under prolonged stress. Over the past 2 years (since October 7th, 2023), Israeli society has faced sustained exposure to prolonged war and armed conflicts, that have subjected the population to acute and chronic stressors including loss, displacement, and a persistent sense of threat (37, 38), resulting in an environment marked by ongoing uncertainty (1, 39). Such a pervasive environment of collective trauma may exacerbate traditional gender differences or, conversely, create a common baseline of distress that levels these variations. Despite the established literature on differences between women and men in mean levels of individual variables (e.g., anxiety symptoms and coping strategies), limited research has examined, particularly in the context of prolonged war, whether the cross-sectional patterns of association among these variables differ between participants reporting female vs. male sex.

Emerging evidence suggests that psychological responses under prolonged war exposure may vary not only in intensity but also in patterns of network organization (40, 41), indicating that differences between women and men may extend beyond isolated mean-level differences in individual variables. Prior trauma-network research suggests that patterns of association among psychological symptoms may vary across samples and contexts, although formal comparisons of overall network structure have often yielded non-significant findings (42, 43). In a recent study conducted among Israeli adults during the current conflict, Spivak-Lavi et al. (44) reported significant differences between women's and men's PTSD-related networks in overall structure and global strength. Their findings suggest that associations between PTSD symptoms and behavioral dysregulation may differ across groups; however, the observed patterns were cross-sectional and do not establish the mechanisms underlying such differences. Taken together, this literature supports an exploratory examination of whether patterns of association differ between participants reporting female vs. male sex during prolonged war exposure. A network approach can describe these cross-sectional patterns of conditional association, while avoiding assumptions about temporal, causal, or gender-specific mechanisms.

Our aim in the current exploratory study was to examine cross-sectional patterns of association among traumatic life events, stress responses (anxiety and depression), health-related quality of life (physical and mental), meaning in life, and coping strategies during prolonged war. Using network analysis, we compared networks estimated separately for participants reporting female vs. male sex. The primary analyses examined whether overall network structure and global strength differed between the two groups. We additionally explored group-specific descriptive patterns in centrality, community organization, and multi-step connectivity. By moving beyond mean-level comparisons, we sought via this system-level perspective to provide a more comprehensive understanding of gender-specific psychological adaptation to chronic adversity using cross-sectional data. Given the exploratory nature of this study, no *a priori* hypotheses were registered in advance.

## Methods and materials

### Participants

The sample comprised 313 adult participants, of whom 159 were women (50.8%) and 154 were men (49.2%), ranging in age from 21 to 80 years (*M* = 43.49, *SD* = 14.13). Most participants were married (60.7%, *n* = 190) with an average of 1.41 children (*SD* = 1.54). Over half of the sample held a higher degree (55.6%, *n* = 174), and 66.1% (*n* = 207) reported an average or below-average income. In terms of religiosity, the majority identified as secular (57.2%, *n* = 179) and a smaller proportion identified as traditional (24.9%, *n* = 78). Geographically, participants resided in the south (38.0%, *n* = 119), the north (32.9%, *n* = 103), and the center (29.1%, *n* = 91) of Israel.

Regarding background characteristics' differences between men and women, there was a nominal difference in education at the uncorrected level. However, none of the differences in age, marital status, education, or household income remained significant after controlling for the false discovery rate (FDR) at 5%, indicating no reliable gender differences in background measures.

### Measures

Participants completed a battery of standardized self-report questionnaires that had previously been used in Israeli populations and demonstrated acceptable psychometric properties.

*Sociodemographic characteristics* included participants' age, sex, marital status, number of children, level of education, religiosity, and region of residence. Regarding sex, participants were asked in Hebrew, “What is your sex?”, with binary self-report response options (male or female). This item did not distinguish between reported sex and gender identity, did not assess gender-related social or cultural processes, and did not provide a non-binary response option.

*Traumatic life events* were assessed via the List of Threatening Experiences Questionnaire (LTE-Q) (45). This instrument consists of 12 highly threatening adverse events (e.g., the death of a family member or close friend) experienced over the past 2 months (46). Items are scored dichotomously (*yes/no*) based on the occurrence of each event. In the current study, Cronbach's alpha was 0.72 (original alpha = 0.84) (33). Additionally, participants were asked about their exposure to traumatic events following October 7th, 2023, and the subsequent multi-front war. This exposure was assessed via three yes/no questions regarding whether they had: (i) lost a family member or close friend, (ii) had a family member or close friend injured, or (iii) had a relative abducted.

*Depression and anxiety* (stress symptoms) were assessed using the Patient Health Questionnaire-4 (PHQ-4) (47). The PHQ-4 is a brief self-report measure comprising two items assessing depression and two items assessing anxiety, referring to symptoms experienced during the past 2 weeks. Items are rated on a 4-point scale ranging from 0 (*not at all*) to 3 (*nearly every day*), yielding a total score ranging from 0 to 12. Cronbach's alphas in the current study were 0.85 for depression and 0.86 for anxiety (original alphas = 0.85 and 0.81, respectively) (47).

*Health-related quality of life* was assessed using the Short Form-12 Health Survey (SF-12) (48), which evaluates physical and mental well-being over the past 2 weeks. The SF-12 yields two summary scores. The physical component summary (PCS) covers physical functioning, role limitations due to physical health, bodily pain, and general health perceptions. The mental component summary (MCS) assesses mental health, vitality, role limitations due to mental health, and social functioning. Responses are weighted and summed to create continuous scales ranging from 0 (*worst*) to 100 (*best*), with scores of 50 or higher indicating better quality of life. In the current study, Cronbach's alphas were 0.79 for the PCS and 0.81 for the MCS.

*Meaning in life* was assessed using the Meaning in Life Questionnaire (MLQ) (29), comprising two subscales: “Presence of meaning” evaluates the extent to which individuals perceive their lives as meaningful (5 items; e.g., “I understand my life's meaning”), and “search for meaning” assesses the active drive to discover or deepen this understanding (5 items; e.g., “I am searching for meaning in my life”). Items are rated on a 7-point scale ranging from 1 (*absolutely untrue*) to 7 (*absolutely true*), with higher scores indicating higher levels of the respective dimension. Cronbach's alphas in the current study were 0.90 for “presence” and 0.88 for “search” (original alphas = 0.86 and 0.87, respectively) (29).

*Coping strategies* were measured using the Brief COPE questionnaire (24), a self-report instrument assessing situational and dispositional coping across 14 two-item subscales. These subscales are grouped into three broad categories: emotion-focused coping (acceptance, emotional support, humor, positive reframing, and religion); problem-focused coping (active coping, instrumental support, and planning); and dysfunctional coping (behavioral disengagement, denial, self-distraction, self-blame, substance use, and venting). Items are rated on a 4-point scale ranging from 1 (“*I haven't been doing this at all”*) to 4 (“*I've been doing this a lot”*). In this study, Cronbach's alphas were 0.73 for emotion-focused, 0.86 for problem-focused, and 0.77 for dysfunctional coping.

### Procedures

Following approval from the institutional review boards of the authors' affiliated institutions, data were collected between November 30 and December 7, 2025, approximately 24–26 months after the onset of the war in Israel on October 7th, 2023. Eligibility criteria included Hebrew-speaking adults aged 21 years or older. Due to the binary response options in the demographic questionnaire, non-binary participants could not be identified or represented as a separate group in the current sample. Participants were recruited through iPanel, Israel's largest online research panel, which includes approximately 100,000 panel members nationwide, operating in accordance with ESOMAR (European Society for Opinion and Marketing Research) international standards. Sampling procedures used predetermined quotas based on sex and geographic region to obtain a broad demographic distribution. Participants received standard panel reward points upon completing the survey. Prior to participation, all eligible individuals were informed about the study's aims and provided explicit informed consent.

### Data analysis

The analyses were conducted on 313 participants with complete data on the grouping variable and all network variables, comprising 154 men and 159 women. Before the primary analyses, the distributions of the ten study measures were examined using Anderson–Darling normality tests. All measures deviated significantly from normality (minimum (*A* = 1.04), (*p* = 0.010)). Multivariate outliers were assessed across the ten network variables using the Minimum Volume Ellipsoid estimator and robust Mahalanobis distances, implemented with the ‘cov.rob' function in the MASS package. Robust distances were compared with the 97.5% chi-square cutoff with ten degrees of freedom. Fifty observations were identified as multivariate outliers, including 24 men and 26 women. These observations were retained in the primary analyses to avoid data-dependent exclusions and preserve the complete analytic sample. The network comparisons were also repeated after excluding these observations as a sensitivity analysis.

We first examined between-group differences in traumatic life events, anxiety, depression, problem-focused coping, emotion-focused coping, dysfunctional coping, physical and mental health-related quality of life, search for meaning, and presence of meaning. Because all measures deviated from normality, the comparisons were conducted using Yuen's robust tests of trimmed means. Robust effect sizes (ξ) were reported for each comparison. Benjamini–Hochberg false-discovery-rate corrections were applied across the ten outcomes. An FDR threshold of 5% was used as the conventional criterion, whereas results at an FDR threshold of 10% were identified explicitly as exploratory.

The network included ten scale-level nodes. Traumatic life events was a bounded count score representing the sum of 12 dichotomously coded event categories from the List of Threatening Experiences Questionnaire (45, 46). The remaining nine nodes were treated as continuous Gaussian variables: the anxiety and depression subscale scores from the Patient Health Questionnaire-4 (47); the problem-focused, emotion-focused, and dysfunctional coping composites from the Brief COPE (24); the physical and mental component summary scores from the Short Form-12 Health Survey (48); and the search-for-meaning and presence-of-meaning subscale scores from the Meaning in Life Questionnaire (29). The network therefore combined one event-count node with nine continuous subscale or composite scores that differed in their number of constituent items, scoring ranges, construct breadth, and measurement precision. Scale- and subscale-level scores were selected because the study aimed to examine conditional associations among broad psychological domains rather than among individual questionnaire items. This approach preserved the established scoring structures of the respective instruments and provided an identical set of conceptually defined nodes for comparison across the two groups. It also limited the dimensionality of the group-specific networks relative to the available sample size, whereas an item-level specification would have required estimating a substantially larger number of parameters and would not have corresponded directly to the study's construct-level research question. Although the nodes differed in breadth and number of constituent items, all scores demonstrated acceptable to high internal consistency in the present sample, with coefficients ranging from 0.72 to 0.90. Notably, the two-item anxiety and depression subscales showed internal-consistency coefficients of 0.86 and 0.85, respectively, indicating that the shorter subscales were not represented by markedly unreliable scores. The same scoring rules and node definitions were applied in both groups, thereby ensuring that the between-group comparisons involved identically operationalized constructs.

All ten nodes were treated as Gaussian variables in the primary partial-correlation networks. The traumatic-life-events count was therefore modeled using a Gaussian approximation rather than a Poisson specification. This provided a common correlation-based estimator for both groups and allowed the ten constructs to be examined within the same network-comparison framework. Because partial correlations are invariant to linear rescaling, differences in the raw units of measurement did not directly determine the edge estimates. In addition, the use of identical node definitions across groups reduced the possibility that between-group differences resulted from different scoring or aggregation procedures. Given the non-normal distributions and the Gaussian treatment of the bounded event-count variable, rank-normalized network estimation was examined in sensitivity analyses to evaluate whether the findings depended on these distributional features. Together, the acceptable reliability of all node scores, the identical operationalization across groups, the scale invariance of partial correlations, and the rank-normalized sensitivity analyses reduced, although they could not eliminate, the influence of differences in measurement precision, aggregation, and distribution. Nevertheless, differences in reliability, aggregation, distribution, and construct breadth may affect attenuation, edge selection, centrality, community assignment, and predictability. The resulting networks should therefore be interpreted as networks of the selected scale-level constructs rather than item-level symptom networks. Given the non-normal distributions and the Gaussian treatment of the bounded event-count variable, rank-normalized network estimation was also examined in sensitivity analyses.

Separate networks were estimated for men and women using the Extended Bayesian Information Criterion graphical least absolute shrinkage and selection operator, or EBICglasso, with a tuning parameter of (\gamma=0.25). The estimator evaluated 1,000 candidate regularization values. Following selection of the network structure, the retained edges were refitted without the LASSO penalty to obtain non-shrunken partial-correlation estimates. Network estimation was conducted using the ‘estimateNetwork' function of the bootnet package.

Edges represent partial correlations between pairs of variables, conditional on all other variables included in the network. The estimated networks consequently represent cross-sectional, between-person patterns of conditional association. They do not establish causal direction, temporal ordering, or within-person psychological processes.

The primary inferential analysis was conducted using the Network Comparison Test implemented in the NetworkComparisonTest package. The analysis used 5,000 participant-label permutations and tested whether the men's and women's networks differed in overall network structure or global strength. Global strength was defined as the sum of the absolute edge weights in each network. The Network Comparison Test also evaluated all 45 individual edge differences and node-specific differences in strength and expected influence. Methodological work distinguishes comparisons of overall network structure, global strength, and specific edge strengths because similarity at one level does not necessarily establish similarity at the others (49).

Strength was defined as the sum of the absolute weights of all edges connected to a node and therefore represented its overall connectivity. Expected influence was defined as the signed sum of the edges connected to a node and represented its aggregate signed connectivity. Expected influence was not interpreted as evidence that a node causally influenced the other variables. Benjamini–Hochberg corrections were applied separately across the 45 edge tests and across the 20 node-by-centrality tests involving strength and expected influence. Closeness and betweenness were also calculated as descriptive shortest-path indices. Because their case-dropping stability was low, they were not included in the primary inferential centrality family and were interpreted only descriptively.

Network density and average local clustering were evaluated separately because the omnibus Network Comparison Test does not directly test every topological characteristic. Density was defined as the proportion of the 45 possible edges receiving non-zero estimates. Average local clustering described the extent to which the neighbors of a node were also connected. Between-group differences in density and average local clustering were tested using 1,000 participant-label permutations. Benjamini–Hochberg correction was applied across these two topological comparisons.

The degree of similarity between the complete network structures was additionally quantified using the Pearson and Spearman correlations between the two vectors of 45 edge weights, cosine similarity, the number of edges selected in both networks, Jaccard overlap among selected edges, and agreement in edge direction among edges selected in both groups. Uncertainty in these similarity indices was assessed using 500 independent-group bootstrap replications. These measures were used to describe the extent of shared architecture and were not interpreted as formal equivalence tests.

Although two ten-node networks may have similar overall structure and global strength, these omnibus properties do not imply that every edge, or every substantively related group of edges, has the same magnitude in both groups. Several modest and directionally consistent edge differences may be concentrated within one part of a network without producing a detectable difference in the network as a whole. Secondary edge-set comparisons were therefore used to determine whether between-group differences were concentrated within particular subsets of theoretically related direct connections. The specific node domains and edge sets contributing to each statistic are identified when the corresponding comparisons are introduced in the Results.

For each edge-set statistic, the relevant direct partial-correlation edges were combined using either their signed values or their absolute magnitudes, depending on whether the analysis concerned the direction of conditional associations or their overall magnitude. The edge-set definitions were fixed before the permutation distributions were generated. Between-group differences were tested using 5,000 two-sided participant-label permutations. At each permutation, group labels were randomly reassigned while preserving the original group sizes, the two EBICglasso networks were re-estimated using the primary specification, and the between-group differences were recalculated. Permutation (*p*)-values were calculated using the absolute observed and permuted differences with a plus-one correction. Benjamini–Hochberg FDR-adjusted (*q*)-values and single-step maxT-adjusted (*p*)-values were calculated across the edge-set comparisons. These analyses were treated as secondary to the omnibus Network Comparison Test.

Community organization was examined using exploratory graph analysis implemented in the EGAnet package. Communities were identified using the Walktrap algorithm applied to graphical-LASSO networks. Community stability was evaluated separately among men and women using 500 non-parametric bootstrap replications. The analyses examined the frequency with which different numbers of dimensions were recovered, the structural consistency of each dimension, the stability of individual node assignments, and the frequency with which pairs of nodes were assigned to the same community.

A focused community-placement analysis examined the extent to which dysfunctional coping was assigned to the same community as the psychological-adjustment variables. When exploratory graph analysis returned an unassigned node, that node was represented as a unique singleton community for the purpose of calculating coassignment statistics. This ensured that an isolated node was scored as not sharing a community with the other nodes rather than producing a missing value. Between-group differences in community placement were evaluated using 1,000 participant-label permutations. The principal statistic represented dysfunctional coping's average coassignment with the psychological-adjustment nodes. Secondary statistics assessed its pairwise coassignment with the individual adjustment variables, its joint assignment with the full adjustment set, and its occurrence as a singleton. Benjamini–Hochberg corrections were applied separately within the secondary placement and pairwise-coassignment families.

Bridge centrality was examined using the networktools package. Bridge strength was defined as the sum of the absolute weights of edges connecting a node to nodes outside its assigned community. One-step bridge expected influence was defined as the signed sum of these cross-community edges. To permit valid between-group comparison, identical community assignments were applied to both networks. The primary common partition was derived from exploratory graph analysis of the full sample after each network variable had been centered within the two groups. Within-group centering removed between-group mean differences before estimation of the common community structure. A theory-aligned three-community partition was also examined as a sensitivity specification.

Between-group differences in bridge strength and bridge expected influence were evaluated using the Network Comparison Test. The primary common-community analysis used 5,000 participant-label permutations, and the theory-aligned sensitivity analysis used 2,000 permutations. For the focal dysfunctional-coping comparison, Benjamini–Hochberg correction was applied across the two bridge metrics. Exploratory all-node results were additionally corrected within each metric across the ten nodes and across the complete set of 20 node-by-metric comparisons. Bridge betweenness and bridge closeness were not included in the inferential analysis because of their dependence on shortest paths and their low stability.

Node predictability was estimated using mixed graphical models implemented in the mgm package. All ten nodes were specified as Gaussian variables, and model selection used the EBIC with a tuning parameter of γ = 0.25. Predictability was expressed as *R*^2^, representing the proportion of variance in each node statistically accounted for by the other nine variables included in its group-specific model. The difference in mean predictability and the ten node-specific predictability differences were evaluated using 1,000 participant-label permutations. Mean predictability was treated as a single test, and Benjamini–Hochberg correction was applied across the ten node-specific comparisons. An absolute difference in *R*^2^ of 0.10 was used only as a descriptive indication of magnitude and not as a criterion of statistical significance. Predictability does not imply that neighboring nodes causally determine a variable, and the remaining variance cannot be attributed to any particular unmeasured social, biological, relational, or environmental factor.

Additional exploratory graph summaries were calculated to characterize connectivity across more than one graph edge. These included cumulative expected influence across paths of one to five edges, two-step bridge expected influence, a five-step decay-weighted connectivity summary using a decay parameter of 0.50, and cumulative connectivity between broader psychological domains across paths of up to three edges. A graph step represented an edge separating two nodes in the estimated network and did not represent elapsed time, psychological transmission, or a temporal transition. These graph-distance measures were treated as descriptive supplementary analyses rather than as independent evidence of between-group differences.

Network topology was also described using small-world indices calculated with the igraph package. Local clustering represented the tendency of connected nodes to form interconnected groups, and average path length represented the average graph distance between pairs of reachable nodes. Small-world characteristics were evaluated by considering clustering and path length jointly. These quantities described the topology of the estimated graphs only and were not interpreted as evidence of information processing, psychological propagation, or resilience.

The accuracy of the estimated edge weights was assessed separately in each group using 1,000 non-parametric bootstrap replications in the bootnet package, following recommendations for assessing sampling variation in psychological networks (50). Bootstrap confidence intervals were used to distinguish comparatively precise edges from edges showing greater sampling variability. The overlap or non-overlap of separate within-group confidence intervals was not used as a substitute for a direct between-group test.

The stability of strength and expected influence was examined using case-dropping bootstraps with 1,000 replications per group. Correlation-stability coefficients represented the maximum proportion of observations that could be removed while retaining, with 95% probability, a correlation of at least 0.70 between the centrality estimates from the complete sample and those from the reduced samples. Shortest-path centrality measures were interpreted cautiously when their stability coefficients were low.

The robustness of the primary Network Comparison Test was examined under several alternative specifications. These included the primary refitted EBICglasso model; a thresholded refitted network; a regularized network without refitting; a non-refitted model using γ =0.50; rank-normalized models using γ = 0.25 and γ =0.50; a thresholded and refitted rank-normalized network; and the primary refitted model after excluding the multivariate outliers. Each specification included a 1,000-permutation comparison of overall network structure and global strength. These analyses evaluated whether the conclusions depended on non-normality, outlier inclusion, regularization strength, refitting, or thresholding.

All inferential tests were two-sided. Unless otherwise stated, (*p* < 0.05) was used as the conventional significance criterion. Nominal results at *p* < 0.10 and Benjamini–Hochberg-adjusted results at *q*<*0.1*0 were identified explicitly as exploratory. Because the study was exploratory and no hypotheses or analytic families were preregistered, the omnibus tests of overall network structure and global strength were distinguished from all secondary edge, edge-set, community, bridge, predictability, and graph-topology analyses.

## Results

## Group differences

Table 1 presents the descriptive statistics and group comparisons between men and women. Following Benjamini–Hochberg false-discovery-rate correction across the ten outcomes at the 5% level, two significant differences emerged. Women reported significantly higher levels of anxiety than did men (*M* = 2.14, *SD* = 1.58 vs. *M* = 1.75, *SD* = 1.72; *p* = 0.004, *q* = 0.022, ξ = 0.22). Similarly, women reported significantly higher emotion-focused coping than did men (*M* = 28, *SD* = 6 vs. *M* = 26, *SD* = 6; *p* = 0.004, *q* = 0.022, ξ = 0.23). Both effects were small to moderate in magnitude.

**Table 1:** Group comparisons by gender.

Characteristic	Men^1^	Women^1^	*p*-value	*q*-value (FDR)^2^	Yuen's *t*^3^	Robust effect size (ξ)^4^
Traumatic life events	1.69 (2.01)	1.46 (1.98)	0.14	0.3	1.48	0.11
Anxiety	1.75 (1.72)	2.14 (1.58)	0.004	0.022	2.90	0.22
Depression	1.82 (1.74)	1.96 (1.65)	0.3	0.5	0.95	0.08
Problem-focused coping	17.91 (5.56)	19.57 (5.34)	0.027	0.068	2.23	0.19
Emotion-focused coping	25.58 (6.20)	27.56 (5.65)	0.004	0.022	2.95	0.23
Dysfunctional coping	15.01 (4.53)	14.92 (4.09)	0.6	0.7	0.51	0.04
Physical quality of life	50.23 (8.19)	50.55 (8.57)	0.7	0.7	0.44	0.04
Mental quality of life	43.76 (11.40)	40.82 (10.31)	0.022	0.068	2.31	0.19
Search for meaning	21.66 (7.12)	22.58 (6.75)	0.3	0.5	0.94	0.08
Presence of meaning	23.73 (7.48)	24.77 (6.26)	0.4	0.5	0.90	0.08

1 Mean (SD).

2 False Discovery Rate (FDR) correction for multiple comparisons.

3 Yuen's test for trimmed means (robust to non-normality and outliers).

4 Robust effect size (ξ) corresponding to Yuen's test.

Two additional differences met the explicitly exploratory 10% FDR criterion. Women reported higher problem-focused coping than did men (*M* = 19.6, *SD* = 5.3 vs. *M* = 17.9, *SD* = 5.6; *p* = 0.027, *q* = 0.068, ξ = 0.19). Additionally, men reported higher mental quality of life than did women (*M* = 44, *SD* = 11 vs. *M* = 41, *SD* = 10; *p* = 0.022, *q* = 0.068, ξ = 0.19).

No statistically significant between-group differences were detected in traumatic life events, depression, dysfunctional coping, physical quality of life, search for meaning, or presence of meaning. Thus, women reported higher anxiety and emotion-focused coping at the conventional 5% FDR level, with exploratory differences in problem-focused coping and mental health-related quality of life at the 10% FDR level. For the remaining outcomes, the analyses did not provide evidence of between-group differences; these non-significant results should not be interpreted as demonstrating equivalence between the groups.

## Network architecture

**Figure 1:**
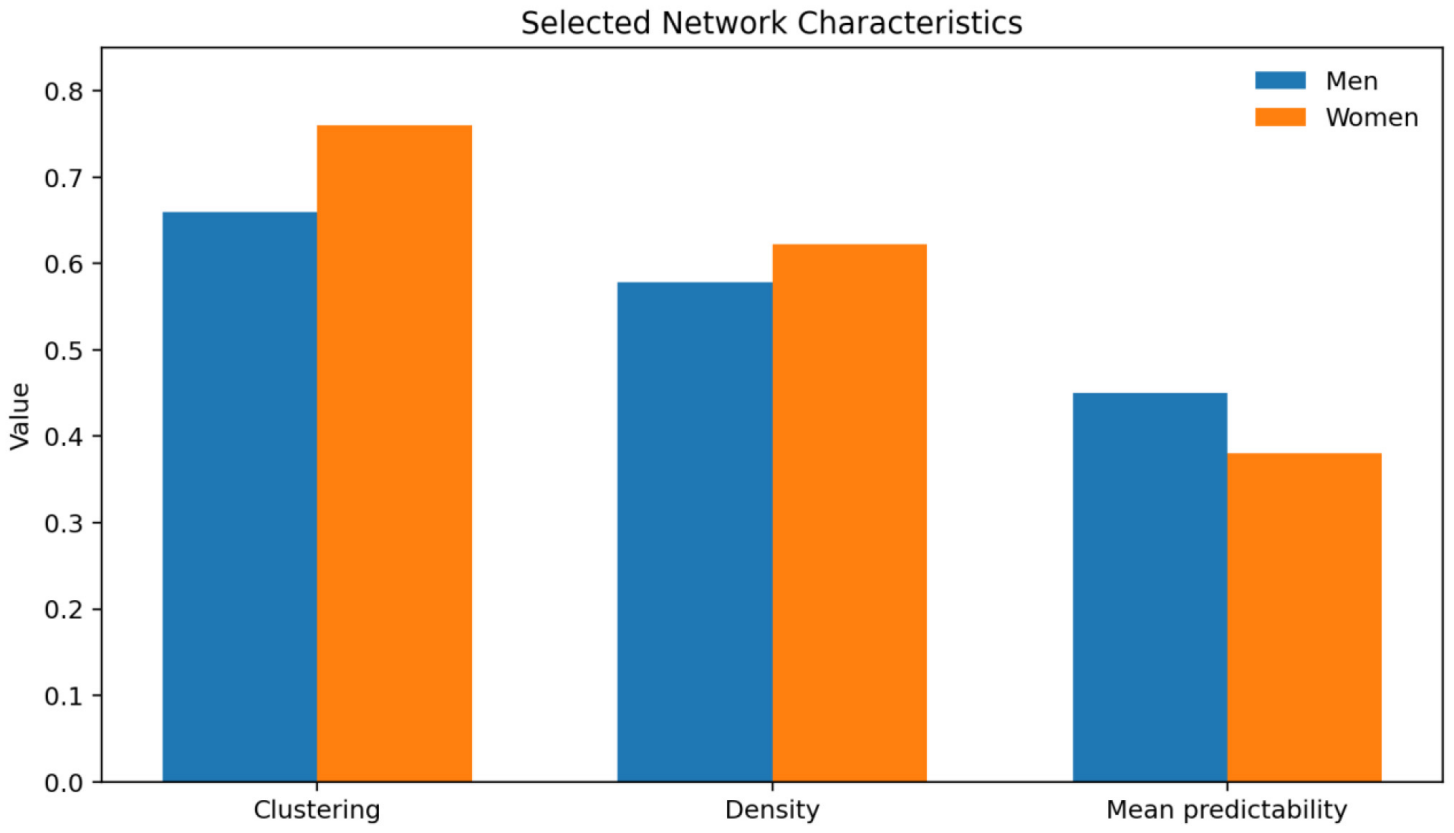
Comparison of selected network characteristics in men and women. The primary Network Comparison Test detected no statistically significant difference in overall network structure (*p* = 0.904) or global strength (*p* = 0.630). Women showed higher density (62.2% vs. 57.8%) and average local clustering (0.76 vs. 0.66), whereas men showed higher mean node predictability (0.45 vs. 0.38). These specific contrasts were evaluated using separate participant-label permutation tests. Bars represent point estimates, and each metric should be interpreted according to its distinct definition.

Figure 1 presents the comparison of network architecture metrics between men and women. Using 5,000 participant-label permutations, the Network Comparison Test revealed no significant differences in global network properties: neither global strength (*p* = 0.630) nor overall network structure (*p* = 0.904) differed significantly between groups, providing no statistically supported evidence of a global difference between the networks. These omnibus Network Comparison Test findings constitute the primary inferential results. As described above, all analyses were based on the same ten predefined scale-level nodes, constructed using the established scoring procedures of the respective instruments and operationalized identically in men and women. The subsequent topology, community, bridge, predictability, and graph-distance analyses were secondary or exploratory examinations of more specific network characteristics. Where direct between-group inference was undertaken, differences were evaluated using participant-label permutation tests rather than visual comparisons of point estimates. Bootstrap procedures were used separately to assess edge-weight accuracy, centrality stability, community stability, and the consistency of node assignments. Benjamini–Hochberg false-discovery-rate corrections were applied within the relevant testing families, and only findings that survived the specified correction were interpreted as statistically supported between-group differences. Findings that did not survive correction, or graph-distance summaries for which no direct inferential comparison was intended, were identified explicitly as descriptive or exploratory.

Despite this overall similarity, descriptive differences were observed in specific metrics. Network density, representing the proportion of possible edges that were present, was slightly higher in women (62.2%) than men (57.8%), indicating that the women's network contained a somewhat greater number of connections between variables. The direct permutation test of the between-group density difference was significant (*p* = 0.046, *q* = 0.046). Conversely, global strength (the sum of all absolute edge weights) was marginally higher in men (4.25) than in women (4.07). This small descriptive contrast was not statistically significant and should not be interpreted as evidence of greater network integration among men.

Small-world properties showed a notable pattern. The women's network demonstrated higher clustering (0.76) than did the men's network (0.66), with the direct permutation test supporting a between-group difference (*p* = 0.032, *q* = 0.046). This indicates that variables in the women's network tended to form more tightly interconnected local clusters. Average path lengths were virtually identical between groups (men: 9.88; women: 9.89), indicating that the average graph distance between variables was similar in the two networks. Mean predictability was higher in men (0.45) than in women (0.38), with the direct permutation test supporting a between-group difference (*p* = 0.041). This indicates that the other variables included in the model accounted for a larger proportion of variance in the nodes of the men's network.

Taken together, these findings suggest that although the two networks did not differ significantly in their global architecture, subtle structural differences emerged: the women's network was slightly denser with higher local clustering, whereas the men's network showed higher node predictability. These patterns may reflect different modes of psychological organization between genders in the aftermath of October 7th.

## Network structure and communities

**Figure 2:**
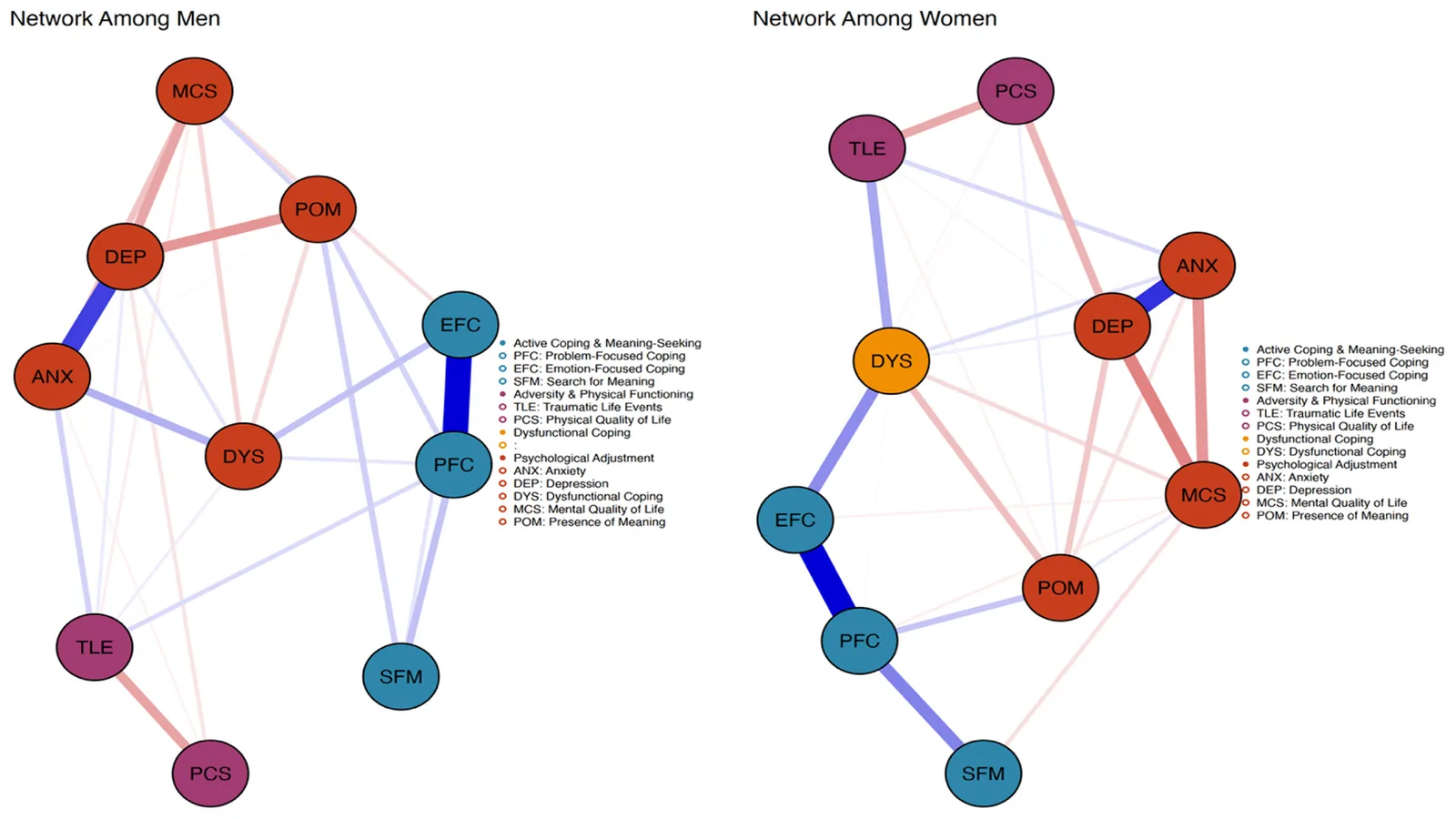
Estimated psychological networks for men (left) and women (right). Nodes represent study variables: TLE, Traumatic Life Events; ANX, Anxiety; DEP, Depression; PFC, Problem-Focused Coping; EFC, Emotion-Focused Coping; DYS, Dysfunctional Coping; PCS, Physical Component Summary of Health-related Quality of Life; MCS, Mental Component Summary of Health-related Quality of Life; SFM, Search for Meaning; POM, Presence of Meaning. Blue edges indicate positive associations, and red edges indicate negative associations, with edge thickness proportional to the strength of the partial correlation. Node colors represent community membership identified by exploratory graph analysis: in the men's network, communities include Active Coping and Meaning-Seeking (blue), Adversity and Physical Functioning (magenta), and Psychological Adjustment (red; notably including dysfunctional coping). In the women's network, communities include Active Coping and Meaning-Seeking (blue), Adversity and Physical Functioning (magenta), Dysfunctional Coping (orange; isolated, not belonging to any community), and Psychological Adjustment (red).

Figure 2 displays the estimated networks for men and women. Exploratory graph analysis using the Walktrap algorithm identified three substantive communities in each network, although dysfunctional coping appeared as an additional isolated node in the women's solution. The women's network contained 28 edges (density = 62.2%), whereas the men's network was slightly sparser with 26 edges (density = 57.8%). In both networks, two communities were identical: (1) an “adversity” and “physical functioning” community comprising traumatic life events and physical quality of life; and (2) an “active coping” and “meaning-seeking” community containing problem-focused coping, emotion-focused coping, and search for meaning.

A difference emerged in how the remaining variables clustered. In the men's network, dysfunctional coping clustered together with anxiety, depression, mental quality of life, and presence of meaning, forming a broad “psychological adjustment” community. This finding indicates that, in the men's estimated network, dysfunctional coping belonged to the same community as anxiety, depression, mental quality of life, and presence of meaning. In contrast, in the women's network, dysfunctional coping was not assigned to one of the substantive communities and appeared as an isolated node. The remaining psychological variables (anxiety, depression, mental quality of life, and presence of meaning) formed their own “psychological adjustment” community without dysfunctional coping.

Bootstrap exploratory graph analysis indicated that a three-dimensional solution was the most frequently recovered structure in both groups, occurring in 61.0% of the men's bootstrap samples and 54.4% of the women's bootstrap samples. A two-dimensional solution was recovered in 37.8% and 41.8% of the respective samples. Dysfunctional coping was assigned with all four psychological-adjustment variables in 82.5% of the men's bootstrap samples and 32.5% of the women's bootstrap samples.

A direct participant-label permutation test supported a statistically significant between-group difference in dysfunctional coping's average co-assignment with the psychological-adjustment variables (*p* = 0.025). The difference in its occurrence as an isolated or singleton node was also statistically significant after correction (*p* = 0.025, *q* = 0.049). Thus, the different placement of dysfunctional coping was evident in the estimated group networks and occurred with different descriptive frequencies across the bootstrap samples and represents a key structural distinction between the networks. Specifically, among men, dysfunctional coping was structurally embedded within the broader psychological-adjustment community. Among women, dysfunctional coping appeared as an isolated node and was less conditionally connected to the variables comprising the psychological-adjustment community. This difference describes the community organization of the two group-level networks and does not indicate how dysfunctional coping operated within individual participants over time.

## Centrality invariance

**Figure 3:**
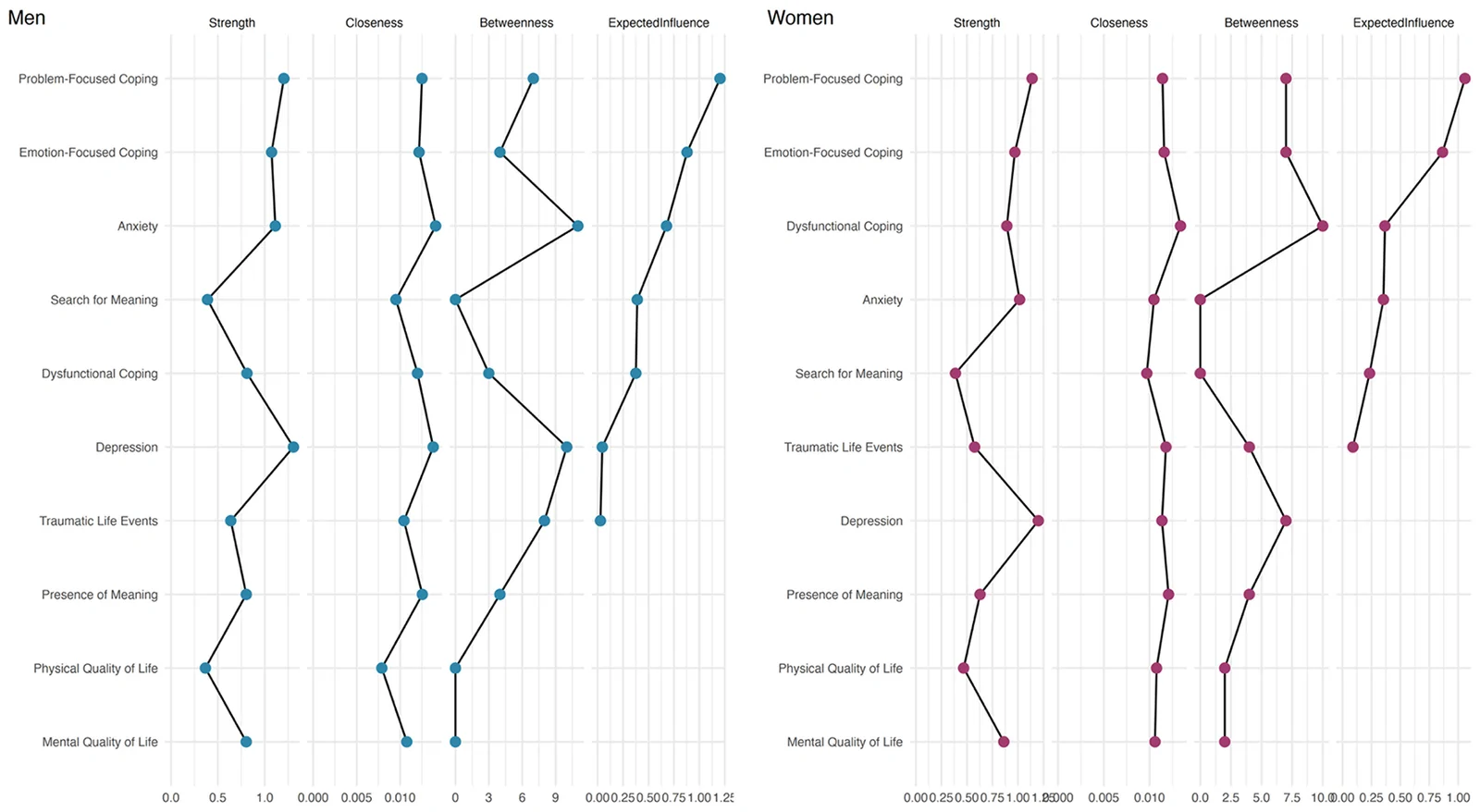
Centrality indices for the men's (left) and women's (right) networks. Strength represents the sum of the absolute edge weights connected to a node, and Expected Influence represents the signed sum of its connected edge weights and therefore its aggregate signed connectivity. No between-group difference in strength or Expected Influence survived Benjamini–Hochberg correction across the 20 node-by-centrality comparisons. Betweenness and closeness are displayed descriptively only because their case-dropping stability was low.

**Figure 4:**
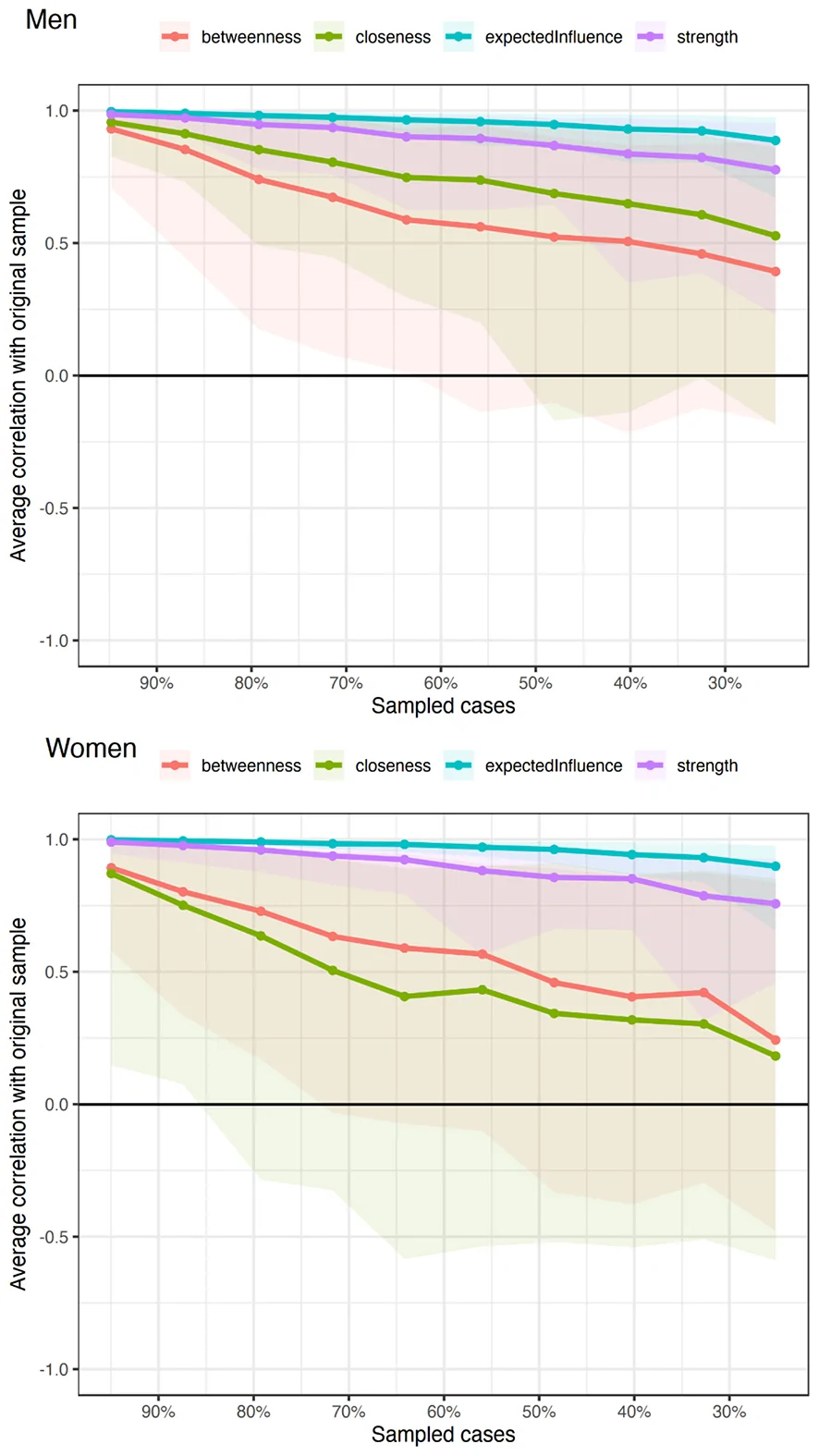
Centrality stability analysis for the men's (top) and women's (bottom) networks based on case-dropping bootstrap with 1,000 iterations. Shaded areas indicate 95% confidence intervals. Expected Influence demonstrated high stability in both networks (CS coefficient: men = 0.75; women = 0.75). Strength was less stable among men than among women (men = 0.36; women = 0.52). Betweenness showed low stability in both groups (men = 0.05; women = 0.05), as did closeness (men = 0.13; women = 0.00); these shortest-path indices were therefore not interpreted substantively.

Figure 3 presents the centrality indices for both networks, and Figure 4 displays the centrality stability analysis. The Network Comparison Test examined differences in strength between-group and Expected Influence using 5,000 participant-label permutations. Benjamini–Hochberg correction was applied across the 20 node-by-centrality comparisons. No centrality difference survived correction; the smallest unadjusted *p*-value was 0.186, and the smallest adjusted *q*-value was 0.927. Thus, the analyses provided no evidence that any node differed between groups in strength or Expected Influence. Betweenness and closeness were retained as descriptive shortest-path indices but were not included in the inferential centrality comparison because of their low stability.

Across both groups, several variables emerged as consistently central. Problem-focused coping exhibited the highest strength and Expected Influence values in both networks, indicating that it had the largest aggregate connectivity with the other included variables in both groups. Emotion-focused coping similarly demonstrated high centrality on these two indices across indices in both groups, reflecting its strong integration within the psychological networks. Conversely, mental quality of life and physical quality of life exhibited comparatively lower strength and Expected Influence values in both networks, suggesting that these health-related quality-of-life variables had lower aggregate connectivity with the other included constructs.

The stability of centrality indices was assessed using a case-dropping bootstrap with 1,000 iterations. Expected Influence demonstrated high stability in both networks (CS-coefficient: men = 0.75; women = 0.75), exceeding the recommended threshold of 0.50. Strength demonstrated lower stability among men than among women (CS-coefficient: men = 0.36; women = 0.52), indicating that the ordering of node-strength estimates was less robust to case removal in the men's network. Betweenness and closeness exhibited low stability in both networks (betweenness CS-coefficient: men = 0.05; women = 0.05; closeness CS-coefficient: men = 0.13; women = 0.00), reinforcing the decision not to interpret these shortest-path indices substantively.

## Bridge centrality and multi-step network connectivity

**Figure 5:**
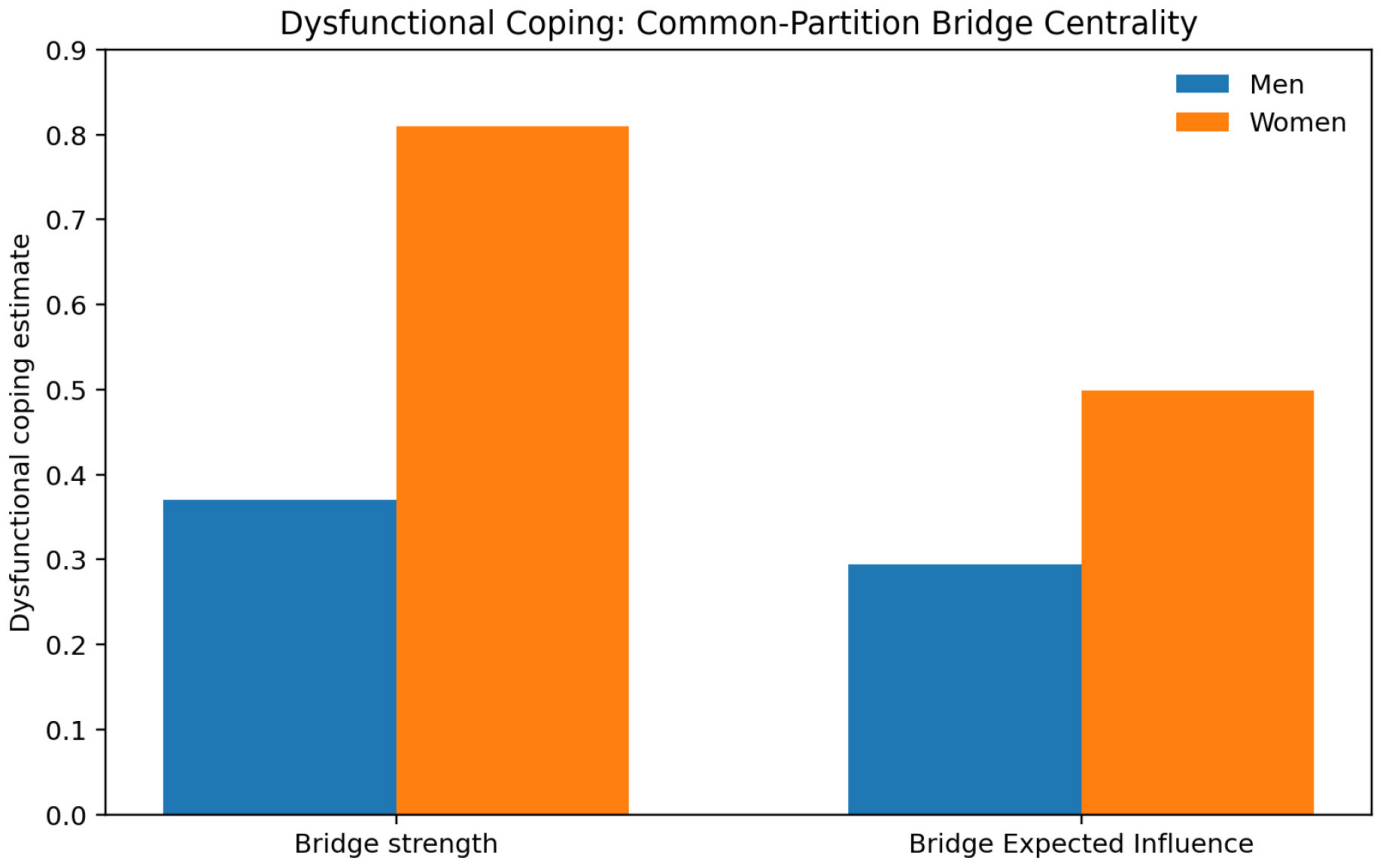
Bridge strength and one-step bridge Expected Influence for dysfunctional coping in the men's and women's networks, calculated using an identical community partition derived from the pooled, within-group-centered data. Dysfunctional coping showed higher bridge strength in women than in men (0.81 vs. 0.37; *p* = 0.005, *q* = 0.037) and higher bridge Expected Influence in women than in men (0.499 vs. 0.294; *p* = 0.007, *q* = 0.037). No other node-level bridge comparison survived correction for multiple comparisons. Bridge indices describe cross-community connectivity within the estimated cross-sectional networks and do not imply causal influence.

To characterize the structural connections among psychological domains, we conducted bridge-centrality analyses and calculated connectivity summaries across multiple graph distances. To ensure that between-group bridge differences were not produced by applying different community assignments to the two networks, bridge centrality was recalculated using the same community partition for men and women. This common partition was derived from exploratory graph analysis of the pooled data after the variables had been centered within groups. Figure 5 presents bridge strength and one-step bridge Expected Influence, and Table 2 presents the direct between-group comparisons.

**Table 2:** Direct between-group comparisons of dysfunctional coping bridge centrality under the common community partition.

Variable	Metric	Men	Women	Difference (Women–Men)	Permutation *p*	FDR-adjusted *q*
Dysfunctional Coping	Bridge strength	0.37	0.81	0.44	0.005	0.037
Dysfunctional Coping	Bridge Expected Influence	0.294	0.499	0.205	0.007	0.037

Identical community assignments were applied to both groups. Between-group differences were evaluated using 5,000 participant-label permutations. Benjamini–Hochberg correction was applied across the two focal bridge metrics. No other node-level bridge comparison survived correction for multiple comparisons.

***Bridge centrality***. Bridge centrality quantifies the extent to which a node is connected to nodes belonging to other communities. Bridge strength represents the sum of the absolute edge weights linking a node to nodes outside its assigned community, whereas bridge Expected Influence represents the signed sum of those cross-community edges. Between-group differences were evaluated using 5,000 participant-label permutations.

Dysfunctional coping showed the largest standardized difference statistic (*z*-equivalent = 3.23), with significantly higher estimated bridge strength in women (*M* = 0.81) than in men (*M* = 0.37; *p* = 0.005, *q* = 0.037). Dysfunctional coping also showed higher bridge Expected Influence in women than in men (0.499 vs. 0.294; *p* = 0.007, *q* = 0.037). No other node-level bridge difference survived correction for multiple comparisons. These common-community analyses distinguish the placement of dysfunctional coping from its cross-community connectivity. The preceding community analysis indicated a significant between-group difference in dysfunctional coping's community assignment, and the bridge analyses additionally provided inferential support for higher bridge strength and bridge Expected Influence among women. Because identical community assignments were applied to both groups, these differences cannot be attributed solely to defining dysfunctional coping as a singleton in the women's network.

***Multi-step network connectivity***. To summarize connectivity beyond immediately adjacent nodes, we additionally calculated cumulative Expected Influence across paths of one to five graph edges, two-step bridge Expected Influence, five-step decay-weighted connectivity, and cumulative connectivity between broader psychological domains across paths of up to three edges. A graph step represents one edge along a network path and does not represent elapsed time, temporal transmission, or within-person psychological change.

The exploratory multi-step analyses showed significantly greater cumulative signed connectivity in the men's network around problem-focused coping, emotion-focused coping, and anxiety. Differences in two-step bridge Expected Influence and decay-weighted connectivity were most apparent for presence of meaning, search for meaning, and mental health-related quality of life, with higher point estimates generally observed among men.

Taken together, dysfunctional coping had higher bridge-centrality point estimates in the women's network under the common community partition. Taken together, dysfunctional coping had significantly higher bridge strength and bridge Expected Influence in the women's network under the common community partition. Thus, dysfunctional coping differed between groups not only in its community placement but also in the magnitude of its cross-community connectivity. In contrast, the exploratory multi-step analyses indicated greater cumulative signed connectivity in the men's network around problem-focused coping, emotion-focused coping, and anxiety, with additional descriptive differences involving meaning in life and mental health-related quality of life. These findings suggest that the women's network was characterized by stronger cross-community connectivity specifically involving dysfunctional coping, whereas the men's network showed greater extended connectivity involving coping and distress-related constructs. Because the multi-step indices summarize paths within undirected, cross-sectional networks, they describe graph connectivity rather than temporal transmission, causal influence, or within-person psychological processes. Full node-level, domain-level, and graph-distance results are presented in Supplementary File 1.

## Edges invariance and edge weight stability

Table 3 presents the Network Comparison Test results examining differences in individual edge weights between the men's and women's networks. The global network structure test indicated that the overall pattern of connections did not significantly differ between groups (*p* = 0.904). Using 5,000 participant-label permutations, all 45 possible edge differences were tested, and Benjamini–Hochberg correction was applied across the complete family of edge comparisons. No individual edge difference remained statistically significant after correction for multiple comparisons.

**Table 3:** Network comparison test of all 45 individual edge-weight differences between men and women.

Variable 1	Variable 2	Permutation *p*	FDR-adjusted *q*

Emotion-focused coping	Search for meaning	0.043	1.000
Traumatic life events	Problem-focused coping	0.097	1.000
Problem-focused coping	Emotion-focused coping	0.119	1.000
Problem-focused coping	Search for meaning	0.128	1.000
Depression	Presence of meaning	0.134	1.000
Anxiety	Dysfunctional coping	0.157	1.000
Dysfunctional coping	Physical quality of life	0.186	1.000
Emotion-focused coping	Dysfunctional coping	0.191	1.000
Traumatic life events	Dysfunctional coping	0.197	1.000
Anxiety	Depression	≥0.200	1.000
Anxiety	Emotion-focused coping	≥0.200	1.000
Anxiety	Mental quality of life	≥0.200	1.000
Anxiety	Physical quality of life	≥0.200	1.000
Anxiety	Presence of meaning	≥0.200	1.000
Anxiety	Problem-focused coping	≥0.200	1.000
Anxiety	Search for meaning	≥0.200	1.000
Depression	Dysfunctional coping	≥0.200	1.000
Depression	Emotion-focused coping	≥0.200	1.000
Depression	Mental quality of life	≥0.200	1.000
Depression	Physical quality of life	≥0.200	1.000
Depression	Problem-focused coping	≥0.200	1.000
Depression	Search for meaning	≥0.200	1.000
Dysfunctional coping	Mental quality of life	≥0.200	1.000
Dysfunctional coping	Presence of meaning	≥0.200	1.000
Dysfunctional coping	Search for meaning	≥0.200	1.000
Emotion-focused coping	Mental quality of life	≥0.200	1.000
Emotion-focused coping	Physical quality of life	≥0.200	1.000
Emotion-focused coping	Presence of meaning	≥0.200	1.000
Mental quality of life	Presence of meaning	≥0.200	1.000
Mental quality of life	Search for meaning	≥0.200	1.000
Physical quality of life	Mental quality of life	≥0.200	1.000
Physical quality of life	Presence of meaning	≥0.200	1.000
Physical quality of life	Search for meaning	≥0.200	1.000
Problem-focused coping	Dysfunctional coping	≥0.200	1.000
Problem-focused coping	Mental quality of life	≥0.200	1.000
Problem-focused coping	Physical quality of life	≥0.200	1.000
Problem-focused coping	Presence of meaning	≥0.200	1.000
Search for Meaning	Presence of meaning	≥0.200	1.000
Traumatic life events	Anxiety	≥0.200	1.000
Traumatic life events	Depression	≥0.200	1.000
Traumatic life events	Emotion-focused coping	≥0.200	1.000
Traumatic life events	Mental quality of life	≥0.200	1.000
Traumatic life events	Physical quality of life	≥0.200	1.000
Traumatic life events	Presence of meaning	≥0.200	1.000
Traumatic life events	Search for meaning	≥0.200	1.000

*p*-values were derived from the Network Comparison Test using 5,000 participant-label permutations. Benjamini–Hochberg correction was applied across all 45 edge comparisons. For edges with unadjusted *p* ≥ 0.200, the table reports the threshold rather than the exact value. No individual edge difference survived correction. Overall network structure: *p* = 0.904.

The emotion-focused coping–search for meaning edge showed the smallest unadjusted *p*-value (*p* = 0.043), followed by the traumatic life events–problem-focused coping edge (*p* = 0.097); however, neither difference survived correction (both *q* = 1,000). Accordingly, these unadjusted results were not interpreted as reliable between-group differences.

This pattern of non-significant edge differences is consistent with the overall network architecture findings showing comparable global structure between groups. The analyses therefore did not identify a specific pairwise conditional association that differed reliably between men and women after accounting for the 45 comparisons. The absence of corrected edge differences does not demonstrate that all corresponding edges were equivalent, but it indicates that no individual difference was statistically supported in the present sample.

Edge-weight accuracy was examined separately in each group using non-parametric bootstrap confidence intervals. In the men's network, 26 of 45 possible edges (57.8%) were non-zero, whereas in the women's network, 28 of 45 possible edges (62.2%) were non-zero. In both networks, the strongest edges demonstrated narrow confidence intervals, indicating greater estimation precision than was observed for weaker edges. The edge between problem-focused coping and emotion-focused coping had the largest estimated weight and one of the narrowest bootstrap confidence intervals in both groups, indicating comparatively high estimation precision in the present sample. This precision does not, by itself, guarantee replication in future samples. The edge between anxiety and depression was also estimated with high precision across both networks, as was the connection between search for meaning and presence of meaning.

Weaker edges showed wider confidence intervals, many of which included zero, indicating substantial uncertainty around these conditional-association estimates. Notably, edges connecting stress symptom variables (anxiety, depression) to health-related quality of life variables (physical, mental) showed negative associations with moderately wide confidence intervals in both networks. The edges connecting dysfunctional coping to other coping strategies showed similar patterns in both groups. Although some corresponding edges differed visually in magnitude, separate within-group confidence intervals were not used to test between-group differences; those comparisons were based on the permutation-based Network Comparison Test reported above.

Overall, the bootstrap results supported comparatively precise estimation of the strongest connections while indicating considerable uncertainty around weaker edges. Both networks showed broadly comparable levels of edge-estimation precision, consistent with their similar group sizes (men, *n* = 154; women, *n* = 159).

## Predictability analysis

**Figure 6:**
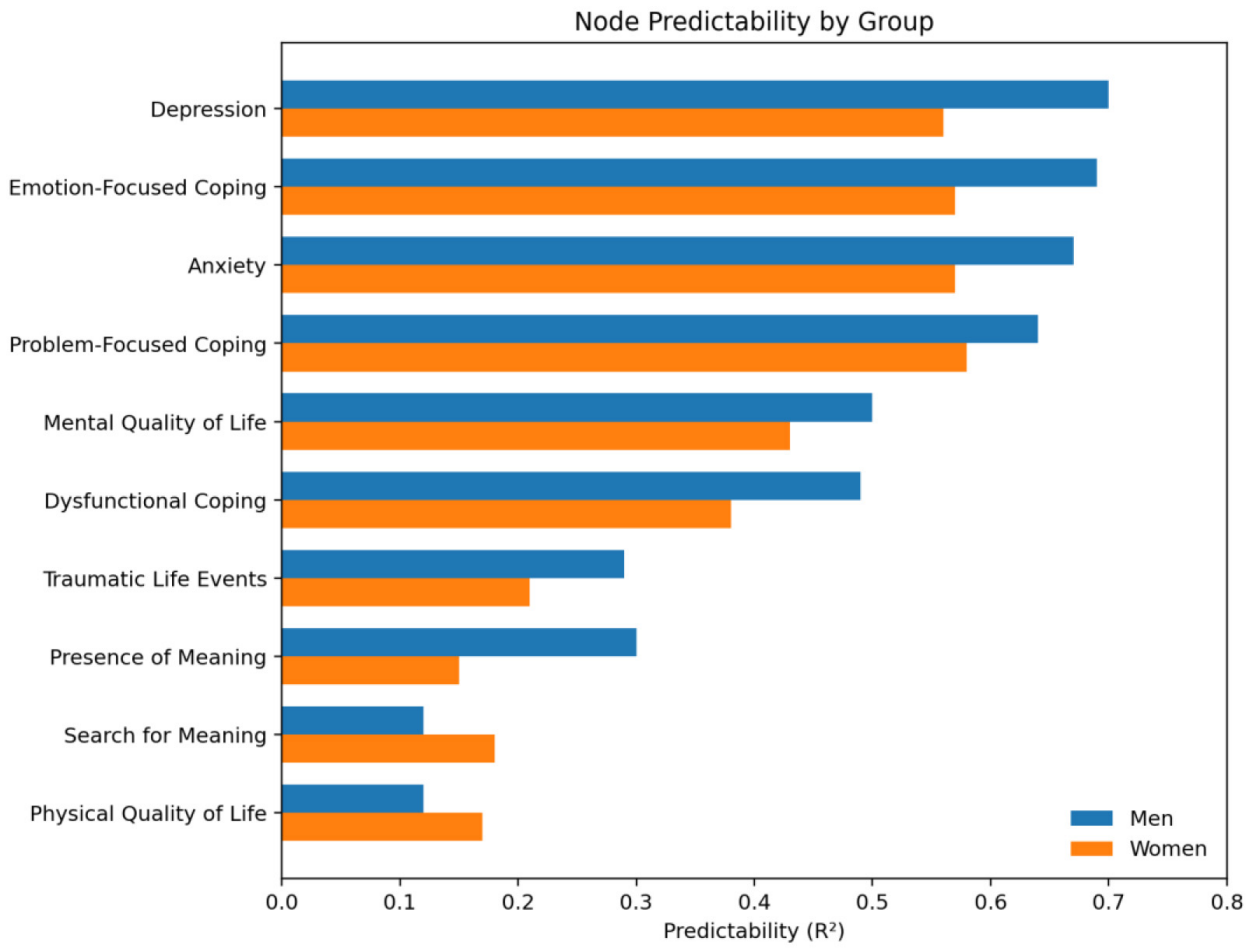
Node predictability (*R*^2^) comparison between men and women, derived from mixed graphical models. Variables are ordered by average predictability across groups. Overall mean predictability was higher in men (*M* = 0.45, SD = 0.22) than in women (*M* = 0.38, SD = 0.18), with a direct participant-label permutation test of *p* = 0.041. Presence of meaning, depression, emotion-focused coping, and dysfunctional coping showed descriptive differences of |Δ*R*^2^| > 0.10, all with higher point estimates in men. This threshold was used only to describe magnitude and was not an inferential criterion; node-specific permutation tests and FDR-adjusted results are reported in Table 4.

Figure 6 presents the node predictability (*R*^2^) values derived from mixed graphical models, indicating the proportion of variance in each node explained by all other nodes in the network. Table 4 presents the full predictability comparison. Overall, mean predictability was descriptively higher in the men's network (*M* = 0.45, SD = 0.22) than in the women's network (*M* = 0.38, SD = 0.18). The direct participant-label permutation test supported this between-group difference (*p* = 0.041), indicating that, on average, the other variables included in the network accounted for a greater proportion of node variance among men.

**Table 4:** Node predictability (*R*^2^) and direct between-group permutation comparisons.

Variable	Men *R^2^*	Women *R*^2^	Difference (Men–Women)	Permutation *p*	FDR-adjusted *q*
Mean predictability	0.45 (0.22)	0.38 (0.18)	0.07	0.041	—
Presence of meaning	0.30	0.15	0.15	0.166	0.553
Depression	0.70	0.56	0.14	0.081	0.415
Emotion-focused coping	0.69	0.57	0.13	0.083	0.415
Dysfunctional coping	0.49	0.38	0.11	0.351	0.568
Anxiety	0.67	0.57	0.10	0.232	0.568
Traumatic life events	0.29	0.21	0.09	0.498	0.568
Mental quality of life	0.50	0.43	0.07	0.463	0.568
Problem-focused coping	0.64	0.58	0.06	0.407	0.568
Search for meaning	0.12	0.18	−0.06	0.511	0.568
Physical quality of life	0.12	0.17	−0.06	0.622	0.622

*R*^2^ values represent the proportion of variance in each node statistically accounted for by the other nine nodes. Mean predictability was evaluated as a single test. Benjamini–Hochberg correction was applied across the ten node-specific comparisons. Differences of |0.10| or greater are descriptive magnitude indicators only and do not constitute significance criteria. Positive differences indicate higher predictability in men. No individual node-specific difference survived FDR correction.

Four nodes showed descriptive between-group differences exceeding the ∣0.10∣magnitude threshold. This threshold was used to summarize the sizes of the observed differences and was not treated as an inferential criterion. Presence of meaning exhibited the largest difference (men: *R*^2^ = 0.30; women: *R*^2^ = 0.15; difference = 0.15), indicating that a larger proportion of variance in presence of meaning was statistically accounted for by the other network variables among men. Depression also showed higher predictability in men (*R*^2^ = 0.70) than in women (*R*^2^ = 0.56; difference = 0.14), indicating that the other included variables accounted for a larger proportion of the variance in depression among men. Emotion-focused coping (men: *R*^2^ = 0.69; women: *R*^2^ = 0.57; difference = 0.13) and dysfunctional coping (men: *R*^2^ = 0.49; women: *R*^2^ = 0.38; difference = 0.11) similarly showed higher predictability in men. The complete node-specific permutation tests and multiplicity-adjusted results are reported in Table 4.

Across both groups, several variables were consistently well-predicted by their network neighbors. Depression demonstrated the highest predictability in men (*R*^2^ = 0.70), whereas emotion-focused coping demonstrated the highest in women (*R*^2^ = 0.57). Anxiety was well-predicted in both networks (men: *R*^2^ = 0.67; women: *R*^2^ = 0.57), as was problem-focused coping (men: *R*^2^ = 0.64; women: *R*^2^ = 0.58). In contrast, physical quality of life and search for meaning exhibited the lowest predictability in both networks (*R*^2^ = 0.12–0.18), indicating that the variables included in the estimated networks accounted for relatively little of their variance. The sources of the remaining variance cannot be identified from the present data.

Taken together, mean predictability was significantly higher in the men's network, with the largest descriptive node-level differences involving presence of meaning, depression, emotion-focused coping, and dysfunctional coping. This pattern is consistent with the greater extended connectivity observed around coping and distress-related constructs in the men's network. Nevertheless, predictability represents statistical shared variance within a cross-sectional network and does not demonstrate causal determination, temporal influence, or the identity of any unmeasured factors contributing to the remaining variance.

## Discussion

The primary objective of this study was to address a central question: do gender-specific patterns of coping and adaptation persist, or do they substantially alter under conditions of chronic, ongoing stress? Drawing on a broad quota-based convenience sample of the Israeli population during the prolonged war that followed the October 7th, 2023, attack on Israel, and guided by the theoretical frameworks of masculine norms and emotion regulation, we framed this investigation as an exploratory study. We highlight that no *a priori* predictions were registered prior to the analyses, allowing us to move beyond traditional mean-level comparisons without assuming temporal or causal directionality. Using network analysis, we examined the underlying cross-sectional architecture of traumatic life events, stress symptoms (anxiety, depression), health-related quality of life, meaning in life, and coping strategies. The primary Network Comparison Test detected no statistically significant difference between men and women in either overall network structure or global strength. Thus, the study did not establish globally distinct psychological network organizations. The subsequent secondary analyses instead examined whether more localized differences were present within networks that were broadly comparable at the omnibus level, including differences in topology, community placement, bridge connectivity, and predictability.

Regarding group differences, women reported significantly higher levels of anxiety and demonstrated greater utilization of emotion-focused and problem-focused coping strategies than did men. These findings align with previous research; specifically, during periods of war and security threats, women have reported higher anxiety than have men (1, 3, 12, 16, 25). Additionally, evidence from both routine and crisis contexts suggests that women are more likely to employ a broader range of coping strategies, including both emotion-focused and problem-focused strategies (27, 41, 51, 52). Interestingly, no significant gender differences were observed in levels of exposure to traumatic life events, depression, dysfunctional coping, or meaning in life.

As an exploratory alternative explanation, the absence of statistically supported differences in overall network structure and global strength may be understood in relation to the concept of “shared traumatic reality” (53). This concept describes circumstances in which entire communities are exposed to pervasive and ongoing threats, potentially reducing distinctions between direct and indirect exposure. Following the October 7, 2023 attack, the Israeli civilian population has experienced sustained collective stress and trauma exposure (2, 37, 39, 54). This context may be relevant to the comparable overall network findings observed in the present sample; however, because exposure characteristics and the processes proposed by the shared traumatic reality framework were not directly assessed, this interpretation remains speculative.

Although previous studies have reported differences between women and men in meaning in life and its associations with psychological outcomes (5, 30, 31), the present study found comparable mean levels of traumatic life events, depressive symptoms, dysfunctional coping, and meaning in life across the two groups. The present null omnibus findings are consistent with a broader literature showing that mean-level differences between men and women do not necessarily translate into statistically significant differences in overall network structure. Formal network comparisons have frequently yielded non-significant structure tests across studies of early post-traumatic stress symptoms, post-terror symptoms, military sexual trauma, depressive symptoms, and pandemic-related stress, including samples substantially larger than the present one (13, 42, 43, 55–57). In particular, Rønning and colleagues (13) reported gender-related differences in early post-traumatic stress symptoms without detecting a statistically significant difference in the overall symptom-network structure.

However, significant formal network differences have also been reported. Spivak-Lavi et al. (44), who examined Israeli adults during the same conflict approximately 4 months after October 7, found significant differences in both overall network structure and global strength. The contrast with the present findings may reflect differences in assessment timing, the constructs represented as nodes, sample composition, exposure characteristics, or other contextual features. Taken together, the available evidence indicates that differences between men's and women's global network organization are neither uniform nor consistently reproduced across samples, measures, and stress contexts. Accordingly, the present null tests of overall structure and global strength should be regarded as the primary findings, while the localized secondary results generate hypotheses concerning more specific aspects of network organization.

Turning to network structure and community detection, both networks showed two comparable communities: one comprising traumatic life events and physical quality of life, and the second containing problem-focused coping, emotion-focused coping, and search for meaning. A descriptive difference between the estimated networks concerned the positioning of dysfunctional coping. In the men's network, dysfunctional coping was grouped within the “*psychological adjustment*” community, alongside anxiety, depression, mental health-related quality of life, and presence of meaning. In the women's network, dysfunctional coping appeared as an isolated node, separate from the primary “*psychological adjustment*” community. Dysfunctional coping, which encompasses maladaptive behaviors such as avoidance, denial, self-blame, and substance use (24), has been associated with poorer quality of life under conditions of prolonged stress (10, 28). In the estimated men's network, its connections with distress, quality of life, and meaning-in-life variables were broadly consistent with prior literature linking avoidant and externalizing coping strategies to distress and maladaptive behavioral outcomes following trauma exposure (58–60).

In the women's network, dysfunctional coping appeared structurally peripheral, forming a separate community, whereas anxiety, depression, mental health-related quality of life, and presence of meaning were grouped within the same community. This descriptive pattern is broadly consistent with Spivak-Lavi et al. (44), who reported dense associations among emotional and behavioral domains in women's PTSD-related network during the current conflict. However, given the cross-sectional design and the absence of statistically supported overall between-group network differences, these patterns should be interpreted as exploratory. In the estimated women's network, dysfunctional coping showed relatively limited connections with distress- and meaning-related variables. Although mean levels of dysfunctional coping were similar among self-identified women and men, this descriptive pattern does not establish that dysfunctional coping was independent of mental health or meaning in life. Emotion-focused coping was more prevalent among women in the present sample; however, the study cannot determine whether emotion-regulation processes account for this pattern. Social support is another possible explanation, given its documented associations with internalizing symptoms (61, 62), but it was not assessed in the present study. Accordingly, these descriptive patterns may generate hypotheses regarding whether dysfunctional coping is associated differently with other variables in the estimated networks of participants reporting female vs. male sex.

Bridge centrality and multi-step connectivity analyses revealed distinct associative patterns within the networks. In the men's network, coping, distress, and meaning-in-life variables showed relatively greater cross-domain connectivity. Specifically, the direct and indirect conditional associations linking coping, distress, and meaning in life were substantially stronger, creating an unbuffered environment where distress was closely coupled with reduced mental quality of life. These findings can be understood by considering traditional masculinity norms alongside emotion regulation frameworks, suggesting that gendered patterns of emotional management may contribute to the persistence or amplification of stress. Because men are often socialized toward restrictive emotionality and self-reliance (63, 64), utilizing maladaptive or avoidant coping during prolonged crises is robustly linked with secondary stressors (65, 66). This forms a detrimental structural coupling (Coping–Distress–Meaning connectivity) that is negatively associated with their meaning in life.

Conversely, the women's network demonstrated a highly modular organization that effectively isolated psychological distress structurally. Dysfunctional coping showed high bridge centrality; however, because this node formed a separate community, this result should be interpreted cautiously. Multi-step connectivity analyses indicated relatively limited cross-domain connectivity involving dysfunctional coping in the estimated women's network. This modularity may have served as a structural buffer, limiting the association between concurrent distress or dysfunctional coping and other central domains. Consequently, women's sense of meaning and mental quality of life remained largely independent of internal symptom connectivity, suggesting their psychological anchors were likely drawn from external, relational factors.

As an exploratory speculation for future research, this pattern can be viewed through the “tend-and-befriend” framework (67) and recent findings on co-rumination. According to Taylor's classic model, women often respond to stress by protecting and nurturing others (“tending”) and seeking out social support networks for mutual defense (“befriending”). During prolonged crises, this intense relational tendency seems to act as a double-edged sword: while excessive emotion-focused coping and social sharing can transition into co-rumination that paradoxically maintains anxiety (68, 69), the “tend-and-befriend” mechanism also deeply embeds their psychological framework within social ties and caregiving roles. Thus, even amid high anxiety within a shared traumatic reality, women's sense of meaning may be externally sustained by these community and family bonds - a hypothesis requiring explicit future testing.

Network predictability analyses showed higher node predictability in the men's network, particularly for depression, presence of meaning, and coping. This pattern indicates that, within the estimated network, these nodes were more strongly associated with the other measured variables, such that coping was more closely interconnected with anxiety, depression, and meaning in life. This pattern is broadly consistent with literature reporting associations between internal distress, coping, and externalizing behaviors among men following traumatic life events (70). Furthermore, the greater structural prominence of internal coping mechanisms in the men's network is broadly consistent with findings reported by Zhang and colleagues (71), who identified emotion regulation as a central node in men's psychological distress networks, whereas women's networks showed greater centrality of external factors, including social support.

Conversely, the women's network exhibited higher local clustering alongside lower overall predictability and weaker cross-domain connectivity (e.g., Coping–Distress). Lower predictability in the women's network means only that the variables included in the present model accounted for a smaller proportion of variance in several women's nodes. It does not identify the source of the unexplained variance. One testable possibility is that adding direct measures of social support and caregiving burden would increase node predictability more strongly among women than among men. This hypothesis should be examined in future research that measures these constructs explicitly. Alternative explanations cannot be ruled out, including greater heterogeneity among the women in the sample, differences in measurement reliability, unmeasured factors, non-linear associations, and sampling variability.

### Limitations

Several limitations should be noted. First, a cross-sectional design restricts causal inference and does not permit the determination of directionality among study variables. Second, the sample may have been subject to selection bias and social desirability. Specifically, the reliance on a broad quota-based convenience sample drawn from an online panel may limit the generalizability of findings to more diverse or underrepresented populations within the broader Israeli society. Notably, our demographic questionnaire did not capture ethnic or national group identity (e.g., Jewish vs. Arab populations). Consequently, our findings cannot disentangle how distinct sociopolitical realities, varying levels of exposure, and different community resources characterizing these specific communities might shape network structures. Additionally, the use of self-report measures may have resulted in underreporting or misreporting, particularly among men, given stigma surrounding emotional expression and adherence to traditional masculine norms (72). Furthermore, while our analyses compared self-identified men and women based on a binary demographic item, our study design cannot isolate the specific biological or socio-cultural mechanisms driving these structural differences. Third, preexisting mental and physical health indicators were not assessed prior to this study, limiting the ability to disentangle observed outcomes from baseline functioning preceding the crisis. Fourth, cultural, contextual, and setting specificity may limit generalizability. The findings reflect local gender norms, military culture, and a specific context of ongoing multi-front conflict following October 7th, 2023, which may operate differently in other sociocultural or national environments. Fifth, our proposition that external factors (such as social support or caregiving burden) explain women's lower network predictability serves as a testable hypothesis for future research rather than a definitive conclusion. Alternatively, the observed lower predictability may simply reflect greater heterogeneity and variance among the women in our sample, without invoking any unmeasured external resources. Finally, the inherent limitations of network analysis must be acknowledged. The estimated network structures are fundamentally conditional on the specific variables included in the model. Furthermore, our models were estimated using cross-sectional data at the between-subjects level. Therefore, the structural findings represent group-level network structures and dynamics, which do not necessarily reflect within-person psychological processes unfolding over time (73).

### 4.2 Implications

The study findings present suggestive descriptive patterns that generate hypotheses for future research regarding interventions during and after prolonged armed conflicts. These observations underscore that gender differences in stress symptoms, health-related quality of life, meaning in life, and coping strategies extend beyond mean-level variation to the fundamental architecture of psychological organization. The highly integrated nature of the men's network, characterized by strong Coping–Distress connectivity, suggests that traditional cognitive-behavioral interventions targeting coping strategies could hypothetically demonstrate broader efficacy. In this tightly coupled system, if confirmed by longitudinal data, enhancing adaptive coping and reducing dysfunctional strategies might be associated with generalized structural improvements throughout the network, such as lower concurrent distress and higher meaning (74). Conversely, the modularity of the women's network and the relative structural isolation of dysfunctional coping suggest that modifying coping strategies alone may not be sufficient to concurrently address anxiety or depression. Consequently, we hypothesize that future interventions for women might benefit from being more holistic and multi-targeted. Future studies should explore whether actively mobilizing external resources and context-sensitive community support systems (75) can play a central regulatory role in the structural organization of coping under shared traumatic realities.

## Conclusions

By applying network analysis to the context of prolonged war, this study found no statistically significant difference between self-identified men and women in overall network structure or global strength. Secondary analyses identified localized differences in selected aspects of network topology, community organization, bridge connectivity, and predictability, but these findings should not be interpreted as establishing globally distinct psychological systems. Rather, they generate hypotheses regarding potentially different patterns of organization within broadly comparable cross-sectional networks. Men appear to exhibit a highly integrated system in which dysfunctional coping, distress, and meaning in life are tightly interwoven structural components. In contrast, women seem to display a more modular organization where these domains exhibit greater structural independence, generating hypotheses for future studies regarding their potential reliance on external, systemic resources or reflecting greater intra-group variance. Importantly, formal comparisons of men's and women's psychological networks have yielded heterogeneous results. Several studies have reported non-significant differences in overall network structure, whereas significant differences have been identified in some larger or contextually distinct samples (13, 42–44, 55–57). Differences in assessment timing, population composition, measured constructs, and the nature of the stressor may contribute to this variability. Therefore, future longitudinal research is warranted to unfold how these gender-specific network structures evolve as communities transition from acute collective crisis to long-term recovery.

## Data Availability

The raw data supporting the conclusions of this article will be made available by the authors, without undue reservation.
