# Supplementary material for: Gendered networks of psychological adaptation under prolonged war
Source: Front Public Health. 2026 Sep 8;14:1920728. doi: 10.3389/fpubh.2026.1920728 (PMC13595433; doi:10.3389/fpubh.2026.1920728)
Supplement: Supplementary file 1 [file Data_Sheet_1.docx]

**Supplementary (File 1) – Exploratory Multi-Step Connectivity Analysis**

**Multi-Step Expected Influence**

To summarize connectivity beyond immediately adjacent nodes, we calculated cumulative Expected Influence scores across paths extending up to five graph steps (see Figure 1). Problem-focused coping showed the largest difference (men: 3.98; women: 3.14; difference = -0.83), followed by emotion-focused coping (men: 3.81; women: 3.05; difference = -0.76) and anxiety (men: 2.10; women: 1.45; difference = -0.65). This pattern indicates greater cumulative signed connectivity around coping strategies and anxiety in the men’s estimated network across the examined graph distances. It does not indicate that influence actually propagated over time.

Analysis of two-step bridge Expected Influence showed that presence of meaning had the largest gender difference (men: 0.38; women: 0.03; difference = -0.35). Thus, cumulative signed connectivity involving presence of meaning across paths of two edges was greater in the men’s network than in the women’s network. Search for meaning (difference = -0.15) and mental quality of life (difference = -0.16) showed similar descriptive patterns, with greater two-step connectivity in the men’s network.

**Decay-Weighted Connectivity Simulation**

A five-step, decay-weighted graph simulation (decay = 0.5) showed that the presence of meaning had the largest between-group difference in both its outward connectivity score (difference = -0.18) and inward connectivity score (difference = -0.26). Mental quality of life also had a lower connectivity score in the women’s network (difference = -0.19). These algorithmic values indicate weaker decay-weighted connectivity involving meaning in life and mental quality of life in the women’s estimated network. They do not represent observed activation, temporal transmission, or within-person psychological change.

**Domain-to-Domain Connectivity Analysis**

Finally, we quantified cumulative connectivity between psychological domains across paths of up to three edges and compared these values using permutation testing with 1,000 iterations (Table 1). Coping-Distress cumulative connectivity was stronger in the men’s network (1.01) than in the women’s network (0.59; p = .046). This finding indicates a stronger cross-sectional pattern of direct and multi-edge associations between coping and distress variables in men; it does not establish that coping affected subsequent distress. Negative Distress-Meaning cumulative connectivity was larger in absolute magnitude in men (-0.88) than in women (-0.46; difference = 0.42). Distress-Health cumulative connectivity was somewhat more negative in women (-1.62) than in men (-1.34; difference = -0.28). All domain-pair statistics were derived from undirected, cross-sectional networks. The domain labels therefore indicate the variables connected by the graph paths and do not specify causal direction or temporal ordering.

**Table 1.** *Cumulative Connectivity Between Psychological Domains Across Paths of Up to Three Edges*

| Domain-Pair Connectivity | Men | Women | Difference (Women - Men) |
| --- | --- | --- | --- |
| Coping-Distress | 1.012 | 0.588 | -0.423 |
| Distress-Meaning | -0.883 | -0.462 | 0.421 |
| Distress-Health | -1.340 | -1.620 | -0.280 |
| Trauma-Distress | 0.486 | 0.366 | -0.120 |
| Coping-Health | -0.670 | -0.618 | 0.052 |
| Trauma-Coping | 0.429 | 0.400 | -0.029 |
| Coping-Meaning | 0.742 | 0.759 | 0.017 |
| *Note.* Values summarize cumulative connectivity in undirected, cross-sectional networks. Domain-pair labels do not indicate causal direction, temporal order, or within-person change | | | |

**Figure 1.** Cumulative Expected Influence profiles for anxiety, depression, dysfunctional coping, and problem-focused coping across five graph steps. Lines represent cumulative Expected Influence at each graph distance, with blue indicating men and magenta indicating women. Problem-focused coping showed greater cumulative signed connectivity in men across the examined graph distances. Anxiety also showed greater cumulative signed connectivity in men, whereas dysfunctional coping showed similar values at one graph step but greater connectivity in men beginning at two graph steps. Graph steps represent numbers of edges along network paths and do not represent temporal intervals or within-person change.
